# Interobserver Agreement for Single Operator Choledochoscopy Imaging: Can We Do Better?

**DOI:** 10.1155/2014/730731

**Published:** 2014-10-08

**Authors:** Amrita Sethi, Theodore Doukides, Divyesh V. Sejpal, Douglas K. Pleskow, Adam Slivka, Douglas G. Adler, Raj J. Shah, Steven A. Edmundowicz, Takao Itoi, Bret T. Petersen, Frank G. Gress, Monica Gaidhane, Michel Kahaleh

**Affiliations:** ^1^Division of Digestive and Liver Diseases, Columbia University Medical Center, New York, NY 10032, USA; ^2^Division of Gastroenterology, North Shore-LIJ Health System, Manhasset, NY 11030, USA; ^3^Division of Gastroenterology, Beth Israel Deaconess Medical Center, Boston, MA 02215, USA; ^4^University of Pittsburgh Medical Center, Pittsburgh, PA 15213, USA; ^5^Gastroenterology, University of Utah SOM, Salt Lake City, UT 84119, USA; ^6^University of Colorado Hospital, Denver, CO 80045, USA; ^7^Department of Internal Medicine/Division of Gastroenterology, Washington University School of Medicine, Saint Louis, MO 63110, USA; ^8^Department of Gastroenterology and Hepatology, Tokyo Medical University, Tokyo 160-0023, Japan; ^9^Department of Gastroenterology and Hepatology, Mayo Clinic, Rochester, MN 55905, USA; ^10^Division of Gastroenterology and Hepatology, Weill Cornell Medical Center, New York, NY 10021, USA

## Abstract

*Background*. The SpyGlass Direct Visualization System (Boston Scientific, Natick, MA) is routinely used during single operator choledochoscopy (SOC) to identify biliary lesions or strictures with a diagnostic accuracy up to 88%. The objective of this study was to determine the interobserver agreement (IOA) of modified scoring criteria for diagnosing biliary lesions/strictures. *Methods*. 27 SPY SOC video clips were reviewed and scored by 9 interventional endoscopists based on published criteria that included the presence and severity of surface structure, vasculature visualization, lesions, and findings. *Results*. Overall IOA was “slight” for all variables. The *K* statistics are as follows: surface (*K* = 0.12, SE = 0.02); vessels (*K* = 0.14, SE = 0.02); lesions (*K* = 0.11, SE = 0.02); findings (*K* = 0.08, SE = 0.03); and final diagnosis (*K* = 0.08, SE = 0.02). The IOA for “findings” and “final diagnosis” was also only “slight.” The final diagnosis was malignant (11), benign (11), and indeterminate (5). *Conclusion*. IOA using the modified criteria of SOC images was slight to almost poor. The average accuracy was less than 50%. These findings reaffirm that imaging criteria for benign and malignant biliary pathology need to be formally established and validated.

## 1. Introduction

The role of cholangioscopy in the diagnosis of biliary strictures is being more widely advocated with increased availability of cholangioscopy systems. The earlier “mother-daughter” systems introduced in the 1970s are being replaced in many practices by the single operator cholangioscopy system (SOCS), which overcomes many of the limitations of these older instruments. The SpyGlass Direct Visualization System (Boston Scientific, Natick, MA) is currently the only SOCS commercially available in the United States. Advantages that this device offers over the older systems include four-way steerability, dedicated irrigation channels, and a 1.2 mm working channel through which diagnostic and therapeutic devices can be used [1, 2]. Peroral video cholangioscopy systems (PVCS) are also available in select Asian countries and on trial basis in the United States and provide superior imaging quality. Common indications for cholangioscopy are stone therapy and evaluation of indeterminate biliary strictures [1–6].

Studies using the SOCS report diagnostic sensitivities for malignancy of 66–78% and specificities of 82–98% [7, 8]. Reports using the PVCS report sensitivity of 100% and specificity of >90% [9]. While the largest multicenter study using SOCS demonstrated high diagnostic accuracy with visualization alone, there was no indication of criteria that were used by the individual investigators in making diagnosis of malignancy [8]. A previous study attempted to establish criteria for the cholangioscopic diagnosis of intraductal pathology by using criteria devised by a single investigator that had allowed for self-assessed accuracy of 90%. When reviewed by multiple observers, however, the interobserver agreement (IOA) of these criteria when viewing video clips was poor [10]. Subsequently, imaging features obtained by PVCS that were felt to be diagnostic of malignancy were published [11]. The objective of this study was to determine the IOA using modified scoring criteria, based on these newly suggested cholangioscopic images, for diagnosing biliary lesions/strictures.

## 2. Methods

Twenty-seven deidentified SPY single operator choledochoscopy video clips and scoresheets were sent out to 9 interventional endoscopists. Each of these endoscopists routinely performs SOC, having performed more than 50 individually. The reviewers were blinded to clinical information related to the indication of the choledochoscopy and the final diagnosis and were not provided correlating fluoroscopic images. The video clips' duration ranged from 1 to 2 minutes. All of the procedures were performed by one expert in choledochoscopy (Michel Kahaleh) over a two-year time period using the same SPY unit. The videos were composed of extracted segments from the procedures and contained images of intraductal strictures and lesions. No video clips were excluded due to poor quality and no annotations or labeling was included in the video clips.

The endoscopists were asked to score the videos using a scoring system based on the criteria published by Itoi et al. in 2009. The clips were scored based on presence and severity of four features: surface structure (flat surface, bumpy surface, and convergence of folds), vasculature visualization (fine network of normal vessels, increased vasculature without bleeding, and increased vasculature with bleeding), lesions (regular granular lesions/hyperplasia, irregularly papillary or granular lesions, and nodular elevated lesions), and findings (normal, inflammation, scar, and cancer). The observers were also asked to choose one of the following final diagnoses: benign, malignant, or indeterminate.

### 2.1. Statistical Analysis

The interobserver agreements for 4 variables and final diagnosis variable were measured using the Fleiss' kappa statistic along with 95% CI. All calculations were performed using SAS version 9.2. *K* statistics were interpreted based on the convention by Landis and Koch: poor agreement, ≤0; slight agreement, 0.01 to 0.20; fair agreement, 0.21–0.40; moderate agreement, 0.41–0.60; substantial agreement, 0.61–0.80; and almost perfect agreement: 0.81–1.00.

## 3. Results

Twenty-seven videos were sent to 9 observers. Forty-one percent (11/27) of the videos featured malignant lesions. Overall interobserver agreement was “slight” for scoring of surface structures (*K* = 0.12, *P* value < 0.0001, and SE = 0.02) and for characterization of vessels (*K* = 0.14, *P* value < 0.0001, and SE = 0.02) and lesions (*K* = 0.11, *P* value < 0.0001, and SE = 0.02). The IOA was also only “slight” for describing findings (*K* = 0.08, *P* value < 0.0001, and SE = 0.03) and for providing a final diagnosis (*K* = 0.08, *P* value = 0.0004, and SE = 0.02).

There was slight to poor agreement on video quality as well. It was felt that there were too few clips to statistically measure accuracy.

## 4. Discussion

It has been shown that direct visualization with the cholangioscopy system improves the accuracy of cholangiographic findings in evaluating patients with biliary obstructive symptoms of indeterminate origin [3, 4, 12–15]. Cholangioscopy also allows for obtaining biopsies under direct visualization. This sampling method has been shown to have sensitivities of 48.9%–76.5%; however, when compared to the sensitivity of a visually based diagnosis, the sensitivity was lower (77.8% versus 48.9%) [8, 13]. The values of a visually based impression may then be to provide accurate diagnosis or to help guide management even in the absence of tissue confirmation of malignancy. Nishikawa et al. reported a 97% accuracy of PVCS imaging of biliary lesions [9]. Recently, Woo et al.'s study showed that the sensitivity, specificity, and overall accuracy of SpyGlass visual assessment and SpyBite biopsy for the diagnosis of malignancy were 100% (21/21) and 64.2% (9/14), 90% (9/10) and 100% (5/5), and 96.7% (30/31) and 73.6% (14/19), respectively [16]. However, both of these studies were performed by single centers. Characteristics of a valid imaging-based diagnostic system are considered to be reproducible and high interobserver agreements when viewed objectively. While diagnostic criteria have been proposed using a video cholangioscopy system, our study demonstrates that the interobserver agreement using the modified criteria for interpretation of cholangioscopic images obtained by a SOC is only slight to almost poor. These findings would suggest that the features of malignant lesions as seen by video cholangioscopy cannot be directly applied to the use of SOCS with satisfactory reproducibility or interobserver agreement. Furthermore, the fair to poor agreement in using these criteria with this cholangioscopy system would suggest that little value should be placed in visual impressions alone and that the diagnostic accuracies reported for choledochoscopy are highly dependent on additional factors such as clinical history and prior ERCP impression and thus may result in the reporting of inflated accuracy levels. Fukuda and others showed that the sensitivity of combined ERCP with cholangioscopy in diagnosing biliary lesions was 93% compared with only 58% for ERCP alone [17]. There was also a superiority of cholangioscopy with biopsy in differentiating benign from malignant lesions with an accuracy of 100% [17].

There are a number of limitations to this study. The most apparent one is that the criteria or features chosen for scoring were not guided by standardized definitions; rather, a scoring system based on the criteria published by Itoi et al. in 2009 was used [11]. Even these criteria had not been validated at the time this study was performed. Another understood limitation is that this is a small retrospective study. The retrospective nature of the study further limits this study in that qualities of the individual optical probes used for the clips cannot be provided nor can standardized length or quality of representative footage be provided. It is interesting to note that when asked to rate the quality of the video, there was only slight to poor agreement. This raises the question of whether measures of quality assurance should be developed and standardized in the future as well.

The results of this study show that interobserver agreements of choledochoscopy images range from slight to almost poor. While there is no doubt that PVCS provide superior imaging to the current fiber optic system used in SOC and provide valuable tools in diagnosing biliary neoplasms, their widespread applicability is limited. In an environment where the current fiber optic system is the dominant system being used worldwide, efforts should be made to establish criteria or revise currently published criteria that are specific to this mode of imaging or that can be consistently accurate across all systems. As with any criteria-based system, these should be validated in further studies. Once established, proficiency in identifying these criteria should be achieved when training to use cholangioscopy for diagnostic purposes.
